# Assessment of occupational exposure to azole resistant fungi in 10 Portuguese bakeries

**DOI:** 10.3934/microbiol.2017.4.960

**Published:** 2017-12-14

**Authors:** Liliana Aranha Caetano, Tiago Faria, Ana Crespo Batista, Susana Viegas, Carla Viegas

**Affiliations:** 1 Environment and Health Research Group (GIAS) Escola Superior de Tecnologia da Saúde de Lisboa, ESTeSL, Instituto Politécnico de Lisboa, Lisbon, Portugal; 2 Research Institute for Medicines (iMed.ULisboa), Faculty of Pharmacy, University of Lisbon, Lisbon, Portugal; 3 Centro de Investigação em Saúde Pública Escola Nacional de Saúde Pública, Universidade Nova de Lisboa, Lisbon, Portugal

**Keywords:** azole-resistance, occupational exposure, bakeries, fungi, *Aspergillus*, Mucorales

## Abstract

Occupational exposure to bioaerosols resulting from handling of flour dust and raw materials in bakeries is associated with health problems. The emergence of azole-resistant fungal species in the environment is thought to be related with the use of azole fungicides in cereal crops and prevention of postharvest spoilage. As raw materials used in bakeries are commonly exposed to azoles, we investigated the mycobiota and azole-resistant fungi prevalence in this occupational environment. Ten Portuguese bakeries were assessed through electrostatic dust cloth (EDC, n = 27), settled dust (n = 7), and raw material (n = 26) samples. Samples were inoculated in malt extract agar (2%) (MEA) with chloramphenicol (0.05 g/L) and in dichloran glycerol (DG18), and onto Saboraud screening media supplemented with 4 mg/L itraconazole, 1 mg/L voriconazole, or 0.5 mg/L posaconazole, and incubated for 3–5 days at 27 °C. Except for one out of the ten analyzed bakeries, *Cladosporium* sp., *Penicillium* sp., and *Aspergillus* sp. were the most prevalent fungi identified. *Aspergillus* sp. and Mucorales order were identified in raw materials with both media, whereas *Penicillium* sp. was identified in DG18 only. Azole-resistant species were identified in the environment (EDC) and, to a lower extent, in raw materials, including *Aspergillus* sp. and Mucorales. The presence of azole-resistant fungal species in bakeries represents an occupational risk for workers. This study proposes complementary sampling methods for the evaluation of occupational exposure to mycobiota, and highlights the importance of studying the prevalence of azole-resistant strains in specific occupational environments.

## 1. Introduction

Handling of flour dust and raw materials in the food industry can be associated with health problems as raw materials may be associated with allergen sensitization and fungal colonization [1]–[6]. Several reports on the relation between fungi levels in diverse occupational environments and health effects [7]–[15] corroborate that fungi are potential occupational health hazards that should be taken into account in risk assessment strategies in occupational settings, including bakeries.

Exposure to flour dust and related bioaerosols in the bakery industry is described to occur mainly during grinding, sifting and mixing operations [16]. When mixing occurs, abundant organic dust particles originating from flour dust disperse into the air and are suspended for a long time before deposited on the floor due to gravitational sedimentation. Consequently, high spreading of fungi and their spores and metabolites, such as volatile organic compounds and mycotoxins, will also probably be suspended [17].

Raw materials used in bakeries consists of finely milled cereal or grains (e.g., wheat, rye, barley, oats, rice, malt, carob, corn) and additional non-cereal ingredients (e.g., enzymes, antioxidants, flavorings and spices, baker's yeast, sugar powder) that are used for dough improvement [16]. Some of these raw materials are ideal microbial growth substrates and can generate elevated levels of bioaerosols [18],[19]. The genus *Aspergillus*, including *A. fumigatus*, is ubiquitous in nature and one of the most prevalent in crops and cereals such as corn, wheat, barley, oat, rice, and sorghum [20].

*Aspergillus* disease affects a broad patient population, from patients with asthma to immunocompromised patients [21]. Invasive fungal diseases, such as aspergillosis, are still a life-threatening complication for immunocompromised patients [22]. Azole drugs are critical in long-term therapy for chronic pulmonary aspergillosis, as they are the only anti-*Aspergillus* agents orally available. This class includes itraconazole (available for clinical use since 1997), voriconazole (since 2002), posaconazole (since 2006), and, most recently, isavuconazole [23],[24]. However, azole resistance has been increasingly reported in both clinical and environmental *Aspergillus* strains [25]–[29].

It is currently discussed whether azole-resistance in environmental strains of *A. fumigatus* can be caused by fungal selection pressure exerted by agricultural triazole fungicide use, such as in crop protection [30], due to the structure similarity of clinical triazoles with triazole fungicides. Azole-resistance mechanisms are increasingly being studied and identified for *Aspergillus* sp., threatening the role of the azole class in the management of fungal diseases [31],[32],[33]. Improved diagnosis and application of fungi-active prophylaxis has led to a reduced incidence of disease due to *Aspergillus* genus. On the other hand, previously rare infectious fungal agents such as *Fusarium* sp. and Mucorales order are on the rise [34],[35],[36]. In this scenario, increasing resistance to the limited arsenal of antifungal drugs is a serious concern, especially for *Aspergillus* and Mucorales infections, for which the therapeutic options have become limited, currently restricted to azoles, echinocandins, polyenes, and flucytosine [37],[38],[39].

Until now, no data regarding exposure to bioaerosols nor azole-resistance distribution in bakeries have been reported for Portugal, and this omission has delayed the application of preventive measures for the protection of workers health. Therefore, the aim of this study was to assess fungal contamination in ten bakeries in Portugal and to determine the prevalence of azole-resistant fungal species in this occupational setting.

## 2. Materials and Method

This study was conducted between May 2016 and June 2017 in 10 Portuguese bakeries located in the Lisbon district and is part of an enlarged exploratory study with financial support from the Portuguese Authority for Working Conditions aiming to characterize occupational exposure to fungi and particles on Portuguese bakeries. While being part of a larger study in which additional environmental characterization was carried out, this paper presents the preliminary results regarding environmental samples collected by passive methods in which azole-resistance monitoring was performed.

### 2.1. Bakeries characteristics

Most bakeries were organized in three different areas: Production—where kneading machines and ovens were located and where dough shaping was performed; Raw material warehouse—where workers collected the raw materials for dough preparation for several times during process; Store—where final product was sold (bread or pastry). In one bakery with no store a distinct area was characterized: Expedition—where distribution of final product for other units occurs. One bakery was dedicated to pastry. The sampling sites and collection periods for each bakery were determined based on the high amount of time spent by workers on those places or dislocation frequency during their occupational activity. In these settings environmental samples (settled dust and electrostatic dust cloth) and several raw materials were collected for the assessment of fungal burden and screening of azole resistance.

### 2.2. Environmental and raw material samples

In total, 34 environmental samples and 26 raw material samples were collected and analysed by culture-based methods (Table 1).

One settled dust sample in each bakery unit (7/10) was collected, by collecting the floor dust into a sterilized bag. After sampling, 4.4 g of the collected floor settled dust were weighted and extracted with 40 mL of distilled water for 20 minutes at 200 rpm, as previously described [40]–[43].

Another approach was used to collect bioaerosols using electrostatic dust cloths (EDCs). This collection device is increasingly being used because it is electrostatic, inexpensive, easy to obtain, and effective at collecting dust [44],[45]. EDCs employ electric fibers which have revealed to increase allergen particle retention [46]. As such, 2–3 EDC samples were collected in each evaluated bakery at distinct working areas, in a total of 27 EDC samples. Each EDC had a surface exposure area of 0.0209 m (19 × 11 cm). The EDCs were placed at a minimum 0.93 m above floor level, and dust was allowed to settle for, at least, 15 days. Each EDC was weighted after sampling and the mean of 10 EDCs weighted before sampling was subtracted. Dust from EDC cloths was extracted with 20 mL 0.9% NaCl with 0.05% Tween80™ by orbital shaking (250 rpm, 60 minutes, at room temperature [40].

**Table 1.** Type and number of samples collected in ten bakeries.

Bakery	Settled dust	EDC	Raw material
1	NA	2	NA
2	NA	3	NA
3	1	2	NA
4	1	2	7
5	1	3	5
6	1	3	5
7	1	3	4
8	1	3	4
9	1	3	NA
10	NA	3	NA

	n = 7	n = 27	n = 26

NA: not applicable.

Twenty six samples of bread/pastry raw material, including different types of flour, sugar and/or spices, were collected (4 to 7 samples per unit) from half of the bakeries evaluated in this study (5 out of 10 units) and prepared as follows: 4.4 g of raw material was weighted and washed with 40 mL of distilled water for 20 minutes at 200 rpm [40]–[43].

### 2.3. Culture-based methods and screening of azole-resistance

The fungal burden was determined in environmental and raw material samples through the inoculation of 150 µL of the wash suspension on 2% malt extract agar (MEA) supplemented with chloramphenicol (0.05%) and dichloran glycerol (DG18). DG18 was used due to its ability to restrict the colony size of fast-growing genera [47], allowing a more complete characterization of fungal growth in complex matrices such as environmental and substrate samples. The prevalence of azole-resistance was determined in all the collected samples using azole-supplemented media by seeding 150 µL of the wash suspension on Saboraud agar supplemented with 4 mg/L itraconazole, 1 mg/L voriconazole, or 0.5 mg/L posaconazole, according to the EUCAST guidelines [48]. All of the collected samples were incubated at 27 °C for 5–7 days, in order to allow the growth of all fungal species present in the samples.

### 2.4. Fungal contamination characterization

After laboratory processing and incubation of the collected samples, quantitative (colony-forming units: CFU/m^2^ and CFU/g) and qualitative results were obtained, with identification of the isolated fungal species or genera. When overgrowth was observed in EDC (>500 CFU) colony count was determined as follows: CFU/(3.14 × area) × dilution factor. In the other samples (settled dust and raw materials), 500 CFU/g was considered as previous applied [41],[42],[43]. For species identification, microscopic mounts were performed using tease mount or Scotch tape mount and lactophenol cotton blue mount procedures. Morphological identification was achieved through macro and microscopic characteristics as noted by De Hoog et al. [49].

### 2.5. Data analysis

The data analysis was performed using univariate descriptive statistics using frequency (n; %), median and graphical representations appropriate to the nature of the data.

## 3. Results

**Figure 1.**
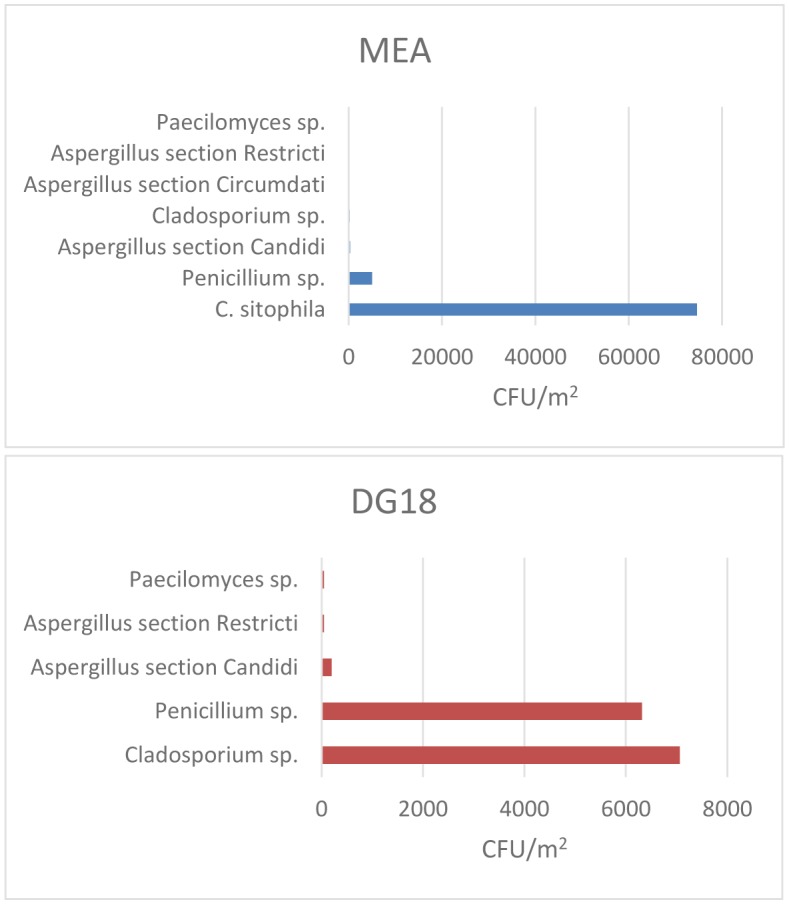
Fungal load in EDC after inoculation onto MEA and DG18 media.

Seven different fungal species were detected in EDC samples from all analyzed bakery units (Figure 1). Considering MEA and DG18 combined (Table 2), *Chrysonilia sitophila* was the predominant species (79.3%), followed by *Penicillium* sp. (12.1%) and *Cladosporium* sp. (7.8%). In addition, *Aspergillus* sp. (0.8%) and *Paecilomyces* sp. (0.1%) were also isolated. Among *Aspergillus* genera, four different species were isolated belonging to three different sections, namely, *Candidi* (0.5%), *Circumdati* (0.2%) and *Restricti* (0.1%) (Table 3). Regarding fungal load distribution among work areas (Table 3), higher fungal counts (CFU/m^2^) were determined either in store/expedition (units n° 5, 6, 8 and 9), production (units n° 3, 4 and 7), or warehouse/packing areas (units n° 1, 2 and 10), with *Penicillium* sp. and *Cladosporium* sp. being the most prevalent species, with one exception (unit n° 10, presenting countless CFU/m^2^ of *Chrysonilia sitophila* at all sampling sites). No fungal growth was detected in settled dust samples.

**Table 2.** Fungal distribution in EDC and raw material samples (fungal count for MEA and DG18 combined).

Fungal species	EDC (CFU*/*m^2^ EDC) (*n*; %)	Fungal species	Raw material (CFU/g) (n; %)
*Chrysonilia sitophila*	74,642; 79.3	*Penicillium* sp.	14; 63.6
*Penicillium* sp.	11,346; 12.1	*Aspergillus* sp.	6; 27.3
*Cladosporium* sp.	7,315; 7.8	Mucorales order	2; 9.1
*Aspergillus* sp.	746; 0.8		
*Paecilomyces* sp.	100; 0.1		

CFU were calculated as follows: (n) = (CFU in MEA + CFU in DG18); (%) = (CFU in MEA + CFU in DG18)/(total CFU in MEA + total CFU in DG18) × 100

Of note, ten different azole-resistant species were identified in 56% (15/27) of the EDC samples from 8 out of 10 assessed bakeries. The most prevalent azole-resistant species were *Chrysonilia sitophila* (49,761 CFU/m^2^ EDC; 64.8%) and *Rhizopus* sp. (Mucorales order) (24,930 CFU/m^2^ EDC; 32.5%), both species not susceptible to 1 mg/L voriconazole, followed by four other azole-resistant species with fungal counts above 100 CFU/m^2^ EDC, namely, *Cladosporium* sp., *Penicillium* sp., *Chrysosporium* sp., and *Aureobasidium* sp., and four other species with lower fungal counts, including *Aspergillus* section *Circumdati*, (Table 4).

**Table 3.** Fungal distribution in different work areas assessed by EDC.

Bakery	Work site	MEA (CFU/m^2^ EDC)	Fungal species	DG18 (CFU/m^2^ EDC)	Fungal species
1	Warehouse	50	*Aspergillus* section *Circumdati*	6,419	*Penicillium* sp., *Cladosporium* sp.
	Production	199	*Aspergillus* section *Candidi*	0
2	Production	0		0	
	Packing	100	*Aspergillus* section *Circumdati*	1,841	*Penicillium* sp., *Cladosporium* sp.
	Store	0		0
3	Warehouse	0		0	
	Production	0		50	*Penicillium* sp.
4	Warehouse	0		0	
	Production	0		199	*Penicillium* sp., *Cladosporium* sp.
5	Production	0		0	
	Warehouse	0		0	
	Store	199	*Penicillium* sp.	100	*Penicillium* sp., *Paecilomyces* sp.
6	Warehouse	0		100	*Penicillium* sp., *Aspergillus* section *Restricti*
	Production	0		0	
	Store	0		149	*Aspergillus* section *Candidi, Cladosporium*
7	Production	199	*Penicillium* sp., *Paecilomyces* sp.	448	*Penicillium* sp.
	Warehouse	100	*Penicillium* sp., *Cladosporium* sp.	199	*Penicillium* sp.
	Store	0		100	*Penicillium* sp.
8	Production	0		50	*Penicillium* sp.
	Warehouse	0		50	*Penicillium* sp.
	Store	3,135	*Penicillium* sp., *Aspergillus* section *Restricti*	2,936	*Cladosporium* sp.
9	Production	249	*Penicillium* sp., *Cladosporium* sp.	299	*Penicillium* sp.
	Warehouse	199	*Cladosporium* sp., *Aspergillus* section *Candidi*	0	
	Store	1,939	*Penicillium* sp.	0
10	Production	24,881	*Chrysonilia sitophila*	100	*Penicillium* sp.
	Warehouse	24,881	*Chrysonilia sitophila*	348	*Penicillium* sp., *Cladosporium* sp.
	Store	24,881	*Chrysonilia sitophila*	299	*Penicillium* sp., *Aspergillus* section *Candidi*

**Table 4.** Azole-resistant fungal species distribution after EDC inoculation onto azole-supplemented Saboraud media.

	EDC (CFU*/*m^2^ EDC) (*n*; %)			

Fungal species	4 mg/L ITC	1 mg/L VRC	0.05 mg/L PSC	Total
*Chrysonilia sitophila*	0; 0	49,761; 65.8	0; 0	49,761; 64.8
*Rhizopus* sp.	0; 0	24,930; 33.0	0; 0	24,930; 32.5
*Cladosporium* sp.	498; 71.4	249; 0.3	249; 55.6	995; 1.3
*Penicillium* sp.	100; 14.3	398; 0.5	149; 33.3	647; 0.8
*Chrysosporium* sp.	0; 0	100; 0.1	50; 11.1	149; 0.2
*Aureobasidium* sp.	50; 7.1	50; 0.1	0; 0	100; 0.1
*Aspergillus* section *Circumdati*	0; 0	50; 0.1	0; 0	50; 0.1
*Paecilomyces* sp.	50; 7.1	0; 0	0; 0	50; 0.1
*Chrysonilia* sp.	0; 0	50; 0.1	0; 0	50; 0.1
*Alternaria* sp.	0; 0	50; 0.1	0; 0	50; 0.1

ITC, itraconazole; VRC, voriconazole; PSC, posaconazole; N, number of species isolates; %, number of species isolates per total of resistant isolates.

**Figure 2.**
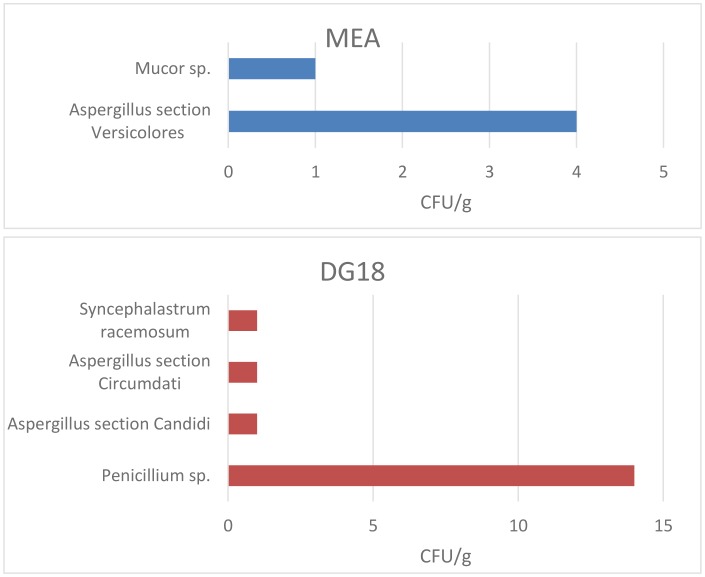
Fungal load in raw material after inoculation onto MEA and DG18 media.

Regarding raw material samples, six different groups of fungal species were isolated (Figure 2). Considering MEA and DG18 combined (Table 2), *Penicillium* sp. was the most prevalent genera (63.6%), followed by *Aspergillus* sp. (27.3%) and *Mucorales* group (9.1%). This fungal contamination was present in 27% (7/26) of the raw material samples collected in four of the five assessed bakeries (Table 5). Among *Aspergillus* genera, four different species were isolated belonging to the sections *Versicolores* (18.2%), *Candidi* (4.5%) and *Circumdati* (4.5%). Among Mucorales, the isolated species were *Mucor* sp. (4.5%), and *Syncephalastrum racemosum* (4.5%). Two azole-resistant fungal species were identified in two distinct raw materials, namely *Chrysosporium* sp. (1 CFU/g) not susceptible to 4 mg/L itraconazole, and *Mucor* sp. (1 CFU/g) not susceptible to 1 mg/L voriconazole (Table 6). No azole-resistant *Aspergillus* species were identified in raw material samples.

**Table 5.** Fungal distribution in raw materials (n = 26) collected at five bakeries (CFU/g).

Raw material ID* (Code)	MEA (CFU/g)	Fungal species	DG18 (CFU/g)	Fungal species
4A	0		0	
4B	0		0	
4C	0		0	
4D	1	*Aspergillus* section *Versicolores*	0	
4E	0		0	
4F	0		0	
4G	0		0	
5E	0		0	
5B	0		0	
5G	1	*Aspergillus* section *Versicolores*	0	
5C	0		0	
5F	3	*Aspergillus* section *Versicolores, Mucor* sp.	1	*Penicillium* sp.
6H	0		0	
6I	0		13	*Penicillium* sp.
6J	0		0	
6K	0		1	*Syncephalastrum racemosum*
6L	0		1	*Aspergillus* section *Candidi*
7M	0		0	
7N	0		1	*Aspergillus* section *Circumdati*
7O	0		0	
7P	0		0	
8B	0		0	
8F	0		0	
8Q	0		0	
8G	0		0	
8E	0		0	

* Code “Number, letter” refers to “Bakery unit, raw material type”.

**Table 6.** Azole-resistant fungal species distribution after raw material inoculation onto azole-supplemented Saboraud media.

	Raw material (CFU*/*g) (*n*; %)			

Fungal species	4 mg/L ITC	1 mg/L VRC	0.05 mg/L PSC	Total
*Chrysosporium* sp.	1; 100			1; 50
*Mucor* sp.		1; 100		1; 50

ITC, itraconazole; VRC, voriconazole; PSC, posaconazole.

## 4. Discussion

This is the first study in Portugal determining the fungal load and prevalence of antifungal-resistant species in bakeries. Overall, with the exception of *Chrysonilia sitophila* for one bakery, the most prevalent fungi isolated in both media were *Cladosporium* sp. and *Penicillium* sp.. Other species with recognized toxigenic potential belonging to the genus *Aspergillus* were also isolated both in the environment and in raw materials.

Exposure to bioaerosols in bakeries may potentially place workers at higher health risk, since exposure to high levels of flour dust potentiates the exposure to airborne microorganisms, which may reach infectious levels within a confined space more readily [3],[7],[16]. In our study, the highest fungal load was found in the production area (1,793 CFU/m^2^ EDC, in MEA and DG18), followed by the warehouse (1,052 CFU/m^2^ EDC, in MEA and DG18) and the store/packing area (957 CFU/m^2^ EDC, in MEA and DG18). Previous studies identified being near the kneading machines during ingredients mixing as the task with higher values for the smallest particles [15],[16]. One possible explanation for this might be related with the use of open machines for mixing without localized exhaustion [15], which is of most importance when, as was the case in the present study, none of the workers used respiratory protection devices.

In the assessment of EDC fungal contamination, different results were obtained using different culture media regarding both fungal load and mycobiota diversity. As an example, the isolates from *Chrysonilia sitophila* were only identified on MEA media. This is due to the ability of DG18 to restrict the colony size of fast-growing genera, such as *Chrysonilia sitophila*, allowing a different and a more complete characterization of fungal contamination. Noteworthy, the use of both media allowed to identify toxigenic genus *Penicillium* and *Aspergillus* (sections *Candidi*, *Circumdati* and *Versicolores*) in the analyzed samples, unveiling a common scenario of potential co-exposure to more than one risk factor—mycobiota and mycotoxins (e.g., aflatoxin) in the baking industry.

Concerning the azole-resistance prevalence in the assessed bakeries, eleven different azole-resistant species, including isolates identified as *Aspergillus* sp. and *Mucor* sp., were detected in the environment and in raw materials. Of note, the number of azole-resistant isolates belonging to the genus *Aspergillus* may be underestimated, in general and in our study, due to the dominance of other genera in the azole-supplemented media with faster growth rates [50]. *Aspergillus* growth restriction can be circumvented by using higher incubation temperatures, as most *Aspergilli* are highly thermotolerant. However, since the evaluation of occupational exposure aims to characterize the complete bioburden, not only *Aspergillus* genera, conventional incubation temperatures were used. The identification of *Mucor* sp. in raw materials is also of concern because invasive fungal diseases due to Mucorales are increasing [34],[35],[36]. Belonging to the order Mucorales, *Mucor* is one of the most commonly identified human pathogenic genera in Europe [51]. Mucorales are not susceptible to voriconazole, the first-line antifungal drug for invasive aspergillosis. The dominant and fast growth of these species in voriconazole screening media may hinder the presence of *Aspergillus* and other species [52].

Although most isolates were not susceptible to 1 mg/L voriconazole only, in four EDC from distinct bakery units three other genus (*Penicillium* sp., *Cladosporium* sp., *Aureobasidium* sp.) were identified as not susceptible to more than one azole. Azole-resistant *Penicillium* and *Cladosporium* spp. were previously reported in clinical isolates for itraconazole and voriconazole [53]. High MICs of voriconazole and isavuconazole were also reported *in vitro* for *Aureobasidium pullulans* in both clinical and environmental isolates [54]. Intrinsic resistance to available antifungals reported in some fungi such as Fusarium, Rhizopus, Rhizomucor and Scedosporium spp. has been pointed out as a major issue by Alhanout and colleagues [55]. However, little is known regarding intrinsic azole-resistance of *Penicillium* sp., *Cladosporium* sp., and *Aureobasidium* sp. In fact, data on intrinsic resistance to azoles are still very limited for non-*A. fumigatus* fungal species. One known example is the intrinsic resistance of *Aspergillus section Terrei* to itraconazole [56]. The fact that overall reported MIC-distributions include only a limited number of clinical isolates for most non-*A.* section *Fumigati* species compared to *A.* section *Fumigati* hinders our ability to distinguish *in vitro* susceptibility at species level. Therefore, molecular identification remains important to gain more insight into the efficacy of antifungal agents [55].

The presence of toxigenic species and azole-resistant species indicates that preventive and protective measures should be implemented to protect both workers' and consumers' health. For instance, most of the *Aspergillus* species from the section *Versicolores* are able to produce sterigmatocystin [57], reported as tumorigenic after oral, intraperitoneal, subcutaneous and/or dermal administration in animals [58]. Also *Aspergillus* section *Circumdati*, and *Penicillium* species can produce ochratoxin A, an hazard for human health due to its carcinogenic, nephrotoxic, hepatotoxic, immunotoxic, and teratogenic effects in animals [59]. One possible measure to reduce fungal burden in this setting would be the use of cleaning products containing fungicides. However, several antifungal substances used as pesticides have been described as potential inducers of azole resistance in environmental *Aspergillus section Fumigati* species. This is due to the fact that fungicides present similar structures to the molecules of clinical azoles [60],[61]. Therefore, it is important to characterize the setting in relation to the prevalence of antifungal resistant species in order to determine which specific biocidals can be used [39],[62].

While the emergence of drug-resistant bacteria such as methicillin-resistant *Staphylococcus aureus* and extensively drug-resistant *Mycobacterium tuberculosis* are already under surveillance policies, it was not until recently that the global problem of antifungal resistance has been recognized as an issue [39]. The increasing occurrence of cryptic species, often drug resistant, as well as of emerging species that are resistant to all antifungal classes [63] illustrates the importance of molecular biology techniques in association with culture-based methodologies [64] for the assessment of occupational exposure to mycobiota [65],[66],[67], as well as for the correct identification of *Aspergillus* species of the section *Fumigati* [68]. This study also corroborates the importance of passive methods (EDC, settled dust and raw material) to complement the exposure assessment. The use of EDC adds information regarding the cumulative presence of bioaerosols in the environment (as they are placed at 1.5 m height and stay in place for 15 days), needing, however, an integrated analysis from the obtained data. It should be pointed out that the main advantage from passive methods is that they can collect contamination from a larger period of time (weeks to several months), whereas air samples can only reflect the load from a shorter period of time (mostly minutes) [69]. Further molecular analyses will be performed in future studies to *Aspergillus* isolates to support a wider project aiming to characterize the prevalence and distribution of *Aspergillus* genera and Mucorales order in different Portuguese occupational environments.

Global warming is increasing the prevalence of crop fungal pathogens, and may also increase the prevalence of fungal disease in humans as fungi adapt to survive in warmer temperatures [70]. It is, therefore, of the outmost importance to perform surveillance studies both in clinical settings and in the environment, including the characterization of azole-resistance prevalence in specific environment compartments (water, soil) and in occupational settings where high fungal load and azole pressure might be expected [67],[71],[72]. International and collaborative efforts are required to understand how resistance develops in the environment to allow effective measures to be implemented aimed at retaining the use of azoles both for food production and human medicine.

## 5. Conclusion

In conclusion, azole-resistant fungal species were detected in Portuguese bakeries. Fungal species resistant to different azoles have been isolated both in environmental samples and in raw materials, including *Aspergillus* sp. and Mucorales. In a context of global azole resistance emerging as a threat to clinical success in the treatment of fungal infections, our results can help in improving prevention. This study provides some approaches to complement conventional exposure assessment process, particularly in highly contaminated occupational settings, with additional sampling (EDC) and screening methods. In order to improve the assessment of occupational exposure to mycobiota and antifungal resistance, both culture-based and molecular methods should be used.
